# Attitudinal variance among patients, next of kin and health care professionals towards the use of containment measures in three psychiatric hospitals in Switzerland

**DOI:** 10.1186/s12888-019-2092-9

**Published:** 2019-04-29

**Authors:** Florian Hotzy, Matthias Jaeger, Etienne Buehler, Sonja Moetteli, Georges Klein, Simone Beeri, Thomas Reisch

**Affiliations:** 1 Department for Psychiatry, Psychotherapy and Psychosomatics, University Hospital of Psychiatry Zurich, Lenggstrasse 31, Postfach 1931, 8032 Zürich, Switzerland; 2 Hospital of Psychiatry Muensingen, Hunzigenallee 1, 3110 Münsingen, Bern, Switzerland; 3 University Hospital of Psychiatry and Psychotherapy, Bern, Switzerland; 4 Département de Psychiatrie et Psychothérapie du Centre Hospitalier du Valais Romand, Route de Morgins 10, 1870 Monthey, Valais Switzerland

**Keywords:** Containment measures, Coercion, Attitudes, Treatment culture, Patients, Next of kin, Health care professionals

## Abstract

**Background:**

In psychiatric treatment containment measures are used to de-escalate high-risk situations. These measures can be characterized by their immanent amount of coercion. Previous research could show that the attitudes towards different containment measures vary throughout countries. The aim of this study was to compare the attitudes towards containment measures between three study sites in Switzerland which differ in their clinic traditions and policies and their actual usage of these measures.

**Methods:**

We used the Attitude to Containment Measures Questionnaire (ACMQ) in three psychiatric hospitals in Switzerland (Zurich, Muensingen and Monthey) in patients, their next of kin (NOK) and health care professionals (HCP). Furthermore, we assessed the cultural specifics and rates of coercive measures for these three hospitals.

**Results:**

We found substantial differences in the usage of and the attitudes towards some containment measures between the three study sites. The study site accounted for a variance of nearly zero in as needed medication to 15% in seclusion. The differences between study sites were bigger in the HCPs’ attitudes (up to 50% of the variance), compared to NOK and patients. In the latter the study site accounted for up to 6% of the variance. The usage/personal experience of containment measures in general was associated with higher agreement.

**Conclusions:**

Although being situated in the same country, there are substantial differences in the rates of containment measures between the three study sites. We showed that the HCP’s attitudes are more associated with the clinic traditions and policies compared to patients’ and their NOKs’ attitudes. One can conclude that patients’ preferences depend less on clinic traditions and policies. Therefore, it is important to adapt treatment to the individual patients’ attitudes.

**Trial registration:**

The study was reviewed and approved by the Cantonal Ethics Commission of Zurich, Switzerland (Ref.-No. EK: 2016–01526, decision on 28.09.2016) and the Cantonal Ethics Commission of Bern, Switzerland (Ref.-Nr. KEK-BE: 2015–00074).

This study has been performed in accordance with the ethical standards laid down in the 1964 Declaration of Helsinki and its later amendments. The permission for conduction of the study was granted by the medical directors at the three study sites. The authors informed the respondents (patients, NOK, HCP) of their rights in the study in an oral presentation and/or a cover letter. They assured the participants of the confidentiality and anonymity of the data, and the voluntariness of participation. Patients were given an information sheet with the possibility to consent in the conduction of the study. Return of the completed questionnaires from HCP and NOK was constituted as confirmation of their consent. No identifying factors were collected to ensure privacy.

This article does not contain any studies with animals performed by any of the authors.

## Background

Psychiatric disorders sometimes can end up in situations in which patients develop such distress that they become a danger to themselves or others. It is one aim of psychiatric emergency treatment to help patients to disrupt such a crisis and prevent them from actual harm against themselves or others. One characteristic of such situations is that they can arise quickly. Because of their potential danger, they have to be controlled fast and safe for the patient, but also for others. Containment measures can be used to break through such situations. They include a variety of interventions which differ in vehemence and force and it is a challenge for health care professionals (HCP) and patients - who should be asked about their preferences whenever possible and even if a coercion has to be used [1] - to choose the methods with the best effect and the least force and coercion on the patient. Containment measures can be categorized by their different grades of coercion. There are some measures in which the grade of coercion seems to be low (e.g. PRN (“pro re nata” = as-needed) medication or intermittent observation) and some where it seems to be strong (e.g. involuntary admission, seclusion, mechanical restraint, coercive medication) [2, 3]. The latter can be summarized as formal coercive measures. Due to the massive restriction of the patients’ freedom coercive measures are regulated by law. Measures from the first category do not go ahead with explicit coercion. Nevertheless, patients have to expect consequences if they reject the offered measure and thus, can also perceive coercion [4].

Containment measures, and especially coercion were shown to be associated with feelings of helplessness [5], humiliation [6] and a reduced satisfaction with therapy in general [7]. Their usage can also lead to avoidance of psychiatric treatment in some patients [8]. Such aversive outcomes were critically discussed since the beginning of modern psychiatry and different stakeholders aimed to strengthen the patients’ autonomy and reduce the number of containment measures - and especially of coercion [9–11]. Nevertheless, until today containment measures are still used in psychiatry in varying frequencies between different countries [12], but also within one country and sometimes even between wards of a single hospital [13, 14].

Previous studies found varying attitudes towards containment measures in patients and HCP [15, 16]. Furthermore, it was shown that not only patients‘ characteristics or preferences but also the traditions and policies of the respective clinics influence the decision for or against specific containment measures [17].

It was shown that higher working experience in HCP was associated with less exertion of coercion [18]. Nevertheless, those HCP who were involved in the exertion of specific coercive measures expressed greater approval towards these measures in one study [6]. Another study found no substantial association between the HCP’s attitudes towards coercion and the actual exertion of coercion [19]. Besides that, it was shown that nurses tend to underestimate higher degrees of coercion compared to physicians [20].

While for most of the HCP the use of coercion seems necessary and justified in some situations, some patients stated that they would have known alternatives to coercive measures but had no possibility to discuss them with the HCP [21]. A high frequency of coercive medication on the ward was associated with more negative attitudes towards containment measures in patients [22]. In contrast, one study found that patients’ personal experiences with coercive medication or seclusion led to higher agreement with the experienced measure [23]. It is suggested that there is not a linear causal relationship between perceived coercion and a specific measure and that some patients perceive different amounts of coercion regarding the same measure [20, 24–26]. These findings illustrate the complexity in which the usage of containment measures is embedded.

The complexity increases if the perspective of next of kin (NOK) is taken into account - as emphasized in recovery-oriented psychiatry. NOK experienced burden from the disorder of the patients [27]. Compared to patients they were more satisfied with the treatment, even if coercion had to be used [28]. To our knowledge, their attitude towards different containment measures with their varying grades of coercion has not been extensively studied yet.

Knowledge about the attitudes of patients, their NOK and HCP towards different containment measures might help to adjust treatment strategies to the patients’ preferences. This should lead to a more founded exertion of these measures in the individual patient with the effect of less perceived coercion [29].

To gain more knowledge about the attitudes towards containment measures against the background of clinic traditions and policies, the aim of this study was to assess and compare the attitudes of patients, next of kin and health care professionals (physicians, psychologists, nurses) at three study sites in different states (cantons) of Switzerland with differing clinic traditions, policies and rates of coercion.

We hypothesized that the study site accounts for a relevant part of the variance in the attitudes of the participants. Furthermore, we hypothesized that the exertion of containment measures at a study site is associated with higher agreement in HCP, patients and their NOK compared to participants from study sites without exertion of these measures.

## Methods

### Setting and legal regulation

We collected data at three study sites in different states of Switzerland. Two (Zurich and Bern) belong to the German speaking part of Switzerland, one (Valais) belongs to the French speaking part of Switzerland. All three study sites are responsible for acute inpatient care and have a public supply mandate. Hence, they are comparable in the diagnoses of their inpatients, the NOK and HCP. Nevertheless, some differences in the clinic traditions and policies can be found. For better illustration the three study sites are described briefly:

### University Hospital of Psychiatry Zurich

The Hospital for Adult Psychiatry of the University Hospital of Psychiatry Zurich provides 220 beds with 14 wards for acute and semi-acute inpatient treatment for patients from 18 to 65 years. With its affiliated departments the clinic constitutes the largest clinic for adult psychiatry in Switzerland, providing mental health services for a catchment area of nearly 500’000 inhabitants from a mostly urban region.

As a university hospital it provides education for medical students, psychologists and nurses and holds different research domains. During the last years the hospital aimed to implement more recovery-based treatment strategies. Also, driven by the high rates of involuntary admissions, alternatives to inpatient treatment have been established including home treatment and treatment of patients in sheltered living units. If a patient has to be constantly observed, the hospital mostly uses external HCP to enable the ward’s HCP for maintaining the clinical routine. The hospital provides an obligatory training in the management of aggression and de-escalation for new HCP from all professions and half year refresher courses for nurses.

### Hospital of psychiatry Muensingen

The Hospital of Psychiatry Muensingen is one of the biggest psychiatric hospitals in Switzerland offering 250 beds. It has 5 specialized wards for acute treatment for adults between 18 and 65 years.

The hospital serves a big catchment area in a rural region with about 500.000 inhabitants.

During the last years, the hospital focused on a reduction of coercive measures and implemented new ward structures with the aim of permanently open wards. There is an obligatory training in the management of aggression and de-escalation for new HCP from all professions. With the development of regularly (one or two times a year) refreshers in de-escalation techniques and management of aggression, the hospital aims to create more awareness for this topic in the future.

Constant observation is provided by the ward HCP. This enables a continuity of the therapeutic relationship.

### Department for Psychiatry and Psychotherapy Monthey

For patients between 18 and 65 years the hospital offers 99 beds. It is located in the French speaking part of Switzerland and serves a rural region with a catchment area of around 250,000 inhabitants. Outpatient alternatives to inpatient treatment are scarce in this area.

The hospital has a long tradition of an open ward policy which started in 1967. At that time, also the rooms for seclusion were abolished and the straps for mechanical restraint were banned. Patients with a potential for endangerment of self or others and a risk to abscond have to wear hospital clothes. This enables easy recognition by the police or general public in the case of their abscondence. This measure is used independently of the legal status of the patient. There are no reliable data on the frequency of this measure. Because of its stigmatizing aspect, the hospital aims for its abolition. The hospital does not transfer patients at risk to other hospitals with exertion of seclusion or mechanical restraint.

There is no structured program of regular HCP training in aggression management and de-escalation. The individual wards are responsible for the training of new colleagues. Constant observation is mostly provided by the ward HCP, seldom by HCP from other wards. This enables a continuity of the therapeutic relationship.

### Mental health legislation in Switzerland

In Switzerland, the exertion of coercion is regulated in the legislation on child and adult protection (Kindes und Erwachsenenschutzrecht, KESR) by the federal civil code which was revised on January 1st, 2013 [30]. Coercive measures (in exactly: orally or intramuscularly administered coercive medication, seclusion, restraint) may be carried out in psychiatric emergency situations “to protect the patient or third parties.” (Art. 435 Swiss Civil Code [30]). They also can be ordered by chief physicians if “failure to carry out the treatment could lead to serious damage to the patient’s health or seriously endanger the life or the physical integrity of third parties” and “the patient is unable to exercise judgement in relation to his or her need for treatment” (Art. 434 Swiss Civil Code [30]).

Involuntary admission (IA) may be executed in “A person suffering from a mental disorder or mental disability or serious neglect (…) if the required treatment or care cannot be provided otherwise.” (Art. 426 Swiss Civil Code [30]). Some procedural aspects regarding IA (in exactly who is authorized to execute an IA and the maximum length of an IA) are regulated differently within some of the 26 states of Switzerland.

### Coercive measures during hospitalization

Next to the description of some organizational structures at the three clinic sites we wanted to compare the actual use of coercive measures at the three study sites. Each study site has a standardized documentation system to record the number and duration of coercion in each patient. Therefore, we analyzed the anonymized routine documentation of coercive measures and police involvement in the case of abscondence which is used for quality control at each study site (see Table 1).

**Table 1:** Characteristics of the three study sites selected

	Area	Bed capacity	Number of admissions	Police involvement at absconding (%)	Ward door policy	Prevalence of coercive measures					
						Rates of involuntary admission (%)	Episodes of Coercion (n)	Episodes of Seclusion (n)	Episodes of Restraint (n)		Episodes of Coercive medication (n)
									physical	mechanical	
Zurich^a^	Urban	220	1699	9.4	Mostly closed	36	514	344	No data	11	159
Muensingen^b^	Rural	251	2632	19.6	Mostly open	29.5	1030	632	No data	203	195
Monthey^b^	Rural	99	1440	8.4	Open^c^	12.6	178	Not used	17	Not used	161

^a^Data from January to June 2016; ^b^Data from 2016, ^c^ Between 07:00 a.m. and 10:00 p.m.

Zurich and Muensingen share some similarities. In comparison, the rate of involuntary admissions in Monthey was lower. Consequent to its policy, seclusion or mechanical restraint were not exerted. However, physical restraint was exerted as an alternative in Monthey; mostly in combination with coercive medication. Mechanical restraint was documented only in a few patients in Zurich whereas in Muensingen its frequency was comparable to the rate of forced medication (oral or intramuscular), which was used in all three study sites. Seclusion was the most frequently used coercive measure in Zurich and Muensingen.

### Study population

The sample consisted of 418 patients (43.5%), 180 next of kin (18.7%) and 364 health care professionals (HCP) (37.8%). We included voluntary and involuntary hospitalized patients with an age > 18 years and < 65 years and all psychiatric diagnoses. Patients with severe cognitive impairment or another condition which forbid informed consent and those with insufficient knowledge of German (in Zurich and Muensingen) or French (in Monthey) language skills were excluded. Of the 418 participating patients 21% were involuntarily hospitalized. Due to the anonymized design we were not able to assess which patients participated or refused. In Zurich 102, in Muenisingen 95 and in Monthey 221 patients completed the questionnaire. Compared to all patients treated at the study sites, the patients included in this study appeared to have a similar distribution of ICD-10 psychiatric diagnoses.

We included first-degree relatives, partners of the patients or a patient’s person of trust if the patients consented to contact them. We defined a maximum of 4 NOK per patient. We excluded a NOK with an age < 18 years and/or if severe cognitive impairment made informed consent impossible or another condition forbid informed consent, or if no informed consent was given. Also, NOK with insufficient knowledge of German (in Zurich and Muensingen) or French (in Monthey) language skills were excluded. In Monthey 47, in Muensingen 80 and in Zurich 53 NOK completed the questionnaire.

We included HCP when they were working for more than 4 weeks at one of the three study sites and had an age > 18 years. They consisted of mental health nurses (*N* = 239 (66%)), psychiatrists (*N* = 90 (25%)) and psychologists (*N* = 31 (8%)). Four (1%) HCP did not define their specialization. Of 257 HCP working at the University Hospital of Psychiatry Zurich 112 (44%) returned the completed questionnaire. At the Hospital of Psychiatry Muensingen 143 (63%) Of 227 HCP, and at the department for Psychiatry and Psychotherapy Monthey 109 (92%) of 118 HCP completed the questionnaire.

### Procedures

Members of the study team informed HCP at the three study sites about the study and directly handed over the anonymized questionnaires. The HCP completed the questionnaires during their working shifts and returned it anonymously.

At each ward, patients were informed about the study by a member of the study team during group meetings. Patients who were interested received information material. Those who gave their informed consent and met the inclusion criteria were included. A member of the study team supported the patients during completion of the questionnaire. The questionnaire used in this study asked for general attitudes towards containment measures. Therefore, patients were included irrespective of their duration of hospitalization (initiation phase, ongoing treatment, termination phase). To achieve a comprehensive sample, each ward was visited at different timepoints.

If the patients gave their consent to invite a NOK, we sent them a letter with the questionnaire and a backward envelope. Due to a low response rate of NOK in the canton of Zurich, we also used relative groups and social media to recruit a sufficient number of NOK.

### Outcomes and measures

We used the German Version of the Attitudes to Containment Measures Questionnaire (ACMQ-D) [31]. For Monthey, the questionnaire was translated to a French version. The translation was conducted by a native German (T.R.) and a native French speaker (G.K) from the study team. The questionnaire is based on the English version developed 2004 by Bowers et al. [3]. The questionnaire fulfills the criteria for usage in research [3] and reached a Cronbachs’ alpha of α = 0.79 to 0.93 in the reliability tests of former studies [32, 33]. It describes 11 containment measures in written form and pictures. The participant can rate the acceptability of each measure with a five-point Likert scale (strongly agree = 1, to strongly disagree = 5). Additionally, the questionnaire contains questions about the respondent’s background (age and gender for patients; education, profession and working experience for HCP; age, education level and profession for NOK), as well as whether the respondent had ever experienced/used the containment measures presented or if the measure was used on the patient related to the NOK.

The ACMQ-D includes a selection of containment measures which are broadly used in the western civilization. Some measures which can be used for containment are not assessed with this questionnaire (e.g. use of walks on the hospital area). On the other hand, some measures are not used in Switzerland (e.g. net bed) but were assessed anyways to assure comparability with other study sites. The measures included are shown in detail in Table 2.

**Table 2:** Definitions of containment methods according to the Attitude to Containment Measures Questionnaire by Bowers et al. (2004) [3]

1	PRN medication	Voluntarily accepted medication administered at the nurses’ discretion in addition to regular doses, by any route
2	Physical restraint	Physically holding the patient, preventing movement
3	Intermittent observation	An increased level of observation, of greater intensity than that which any patient generally receives, coupled with allocation of responsibility to an individual nurse or other worker, periodic checks at intervals
4	Seclusion	Isolated in a locked room
5	Time out	Patient asked to stay in a room or area for a period of time, without the door being locked
6	IM medication	Intramuscular injection of sedating drugs administered without consent
7	PICU	Transfer to a specialist locked ward for disturbed patients
8	Mechanical restraint	The use of restraining straps, belts or other equipment to restrict movement
9	Constant observation	An increased level of observation, of greater intensity than that which any patient generally receives, coupled with allocation of responsibility to an individual nurse or other worker; Constant: within eyesight or arms reach of the observing worker at all times
10	Net bed	Patient placed in a net bed enclosed by locked nets, which he or she is unable to leave
11	Open-area seclusion	Isolated in a locked area, accompanied by nurses

*PRN* Pro re nata, *PICU* Psychiatric intensive care unit, *IM* Intramuscular

### Statistical analysis

We compared differences in attitudes by using univariate ANOVA and Sidac post-hoc tests.

To test for normal distribution of data we used Kolmogorov-Smirnov-Test, Shapiro-Wilk-Test and optical analysis of histograms. We also double-checked results by Kruskal-Wallis test as most variables were only nearly normally distributed. We examined relationships between attitudes and use/personal experience of containment measures by Spearman correlation coefficients. For statistical analyses we used SPSS 23.0 (IBM Corp. Released 2011. IBM SPSS Statistics for Windows, Version 23.0. Armonk, NY: IBM Corp.) for Windows. The level of significance was set at *p* < 0.05.

## Results

### Descriptive statistics

Overall, *n* = 1037 persons participated. Of these respondents, six persons were below the age of 18 years, 54 persons were over 65 years and 13 persons did not indicate their age. Furthermore, two persons were excluded with missing values over 50% in the target outcome variable. The final data set included *n* = 962 individuals of which 318 (33.1%) were from Muensingen, 267 (27.8%) from Zurich and 377 (39.2%) from Monthey. Fifty-four percent (*N* = 521) of the responders were female and gender did not significantly differ between the three study sites. Mean age was 40.1 years (SD = 12.4) and respondents from Zurich were significantly younger (M = 37.0, SD = 11.8) than respondents from Muensingen (M = 42.2, SD = 12.4) and Monthey (M = 40.5, SD = 12.5; F = 13.69 (2959), *p* < .05).

### Global attitudes

We assessed the global attitudes at the three study sites by calculating the mean of all 11 responses for each participant. The mean scale value of global attitude towards containment measures was M = 2.51 (SD = 0.66). We found a good internal consistency with a Cronbach’s alpha =0.83. When we compared the global mean of patients, NOK and HCP we found significant differences between the study sites in HCP and NOK but not within the group of patients (see Table 3). Furthermore, in Muensingen and Zurich, patients showed lower acceptance than NOK and HCP whereas in Monthey, respondents of all groups had comparable values.

**Table 3:** Mean differences in global attitudes of containment methods among patients, relatives and staff, separated by study sites

	Total	Muensingen	Zurich	Monthey	F (df)	p	eta squared
	M (SD)	M (SD)	M (SD)	M (SD)			
Patients	2.77 (0.76)	2.78 (0.74)^d,e^	2.86 (0.81)^e^	2.68 (0.73)	24.68 (8, 953)	0.000	0.172
NOK	2.55 (0.54)	2.38 (0.59)^f^	2.55 (0.48)^f^	2.72 (0.55)			
HCP	2.27 (0.42)	2.07 (0.38)^a,b^	2.14 (0.47)^c^	2.61 (0.40)^a,c^			

*NOK* Next-of-kin, *HCP* Healthcare professionals

^a^Muensingen vs. Monthey, p < 0.05, Sidac post-hoc

^b^Muensingen vs. Zurich, p < 0.05, Sidac post-hoc

^c^Monthey vs. Zurich, *p* < 0.05, Sidac post-hoc

^d^Patients vs. NOK within one study site, *p* < 0.05, Sidac post-hoc

^e^Patients vs. HCP within one study site, p < 0.05, Sidac post-hoc

^f^NOK vs. HCP within one study site, p < 0.05, Sidac post-hoc

### Detailed analysis of the participants’ attitudes at the three study sites

The attitudes varied among the different measures with PRN rated as the most acceptable (M = 1.62; SD = 0.85) and the net bed as the least acceptable (M = 4.10; SD = 1.05) measure (Table 4). Attitudes towards most measures significantly differed among the three study sites, especially between Monthey and the two others. Particularly, in PICU and seclusion the factor of location explained 12 to 15% of the variance in the attitudes of the whole group of participants. When we compared the three participating groups (patients, NOK and HCP) at the three study sites, we found that attitudes towards some containment measures varied among patients, NOK and HCP.

**Table 4:** Mean differences in attitudes of different containment methods among the three study sites, separated by the participating groups

		Total	Muensingen	Zurich	Monthey	F (df)	p	eta squared
Patients		M (SD)	M (SD)	M (SD)	M (SD)			
1	PRN (as-needed) medication	1.81 (0.98)	1.94 (1.00) a	2.17 (1.17) c	1.58 (0.79)	14.48 (2407)	0.000	0.066
3	Intermittent observation	2.04 (1.00)	2.21 (1.07) a	2.29 (1.14) c	1.84 (0.84)	9.32 (2407)	0.000	0.044
5	Time out	2.35 (1.12)	2.38 (1.05)	2.36 (1.11)	2.33 (1.17)	0.10 (2411)	0.908	
9	Constant observation	2.38 (1.07)	2.40 (1.09)	2.45 (1.13)	3.35 (1.03)	0.36 (2410)	0.701	
7	PICU	2.67 (1.21)	2.46 (1.07)	2.54 (1.19)	2.82 (1.27)	3.64 (2408)	0.027	0.018
11	Open-area seclusion	2.53 (1.10)	2.48 (1.14)	2.60 (1.20)	2.51 (1.04)	0.30 (2407)	0.738	
2	Physical restraint	2.73 (1.27)	3.04 (1.28) a	2.91 (1.32) c	2.52 (1.21)	7.11 (2412)	0.001	0.033
6	IM medication	3.06 (1.34)	3.29 (1.30) a	3.46 (1.40) c	2.77 (1.26)	11.57 (2410)	0.000	0.053
4	Seclusion	3.04 (1.30)	2.89 (1.23)	2.80 (1.40) c	3.22 (1.26)	4.44 (2412)	0.012	0.021
8	Mechanical restraint	3.49 (1.28)	3.46 (1.26)	3.68 (1.33)	3.41 (1.25)	1.55 (2407)	0.214	
10	Net bed	4.08 (1.12)	3.98 (1.14)	4.18 (1.18)	4.08 (1.09)	0.76 (2407)	0.468	
NOK								
1	PRN (as-needed) medication	1.85 (0.86)	1.91 (0.85)	2.02 (0.93) c	1.57 (0.75)	3.86 (2176)	0.023	0.042
3	Intermittent observation	1.69 (0.64)	1.66 (0.64)	1.85 (0.57)	1.54 (0.69)	3.00 (2176)	0.052	
5	Time out	2.06 (0.81)	1.89 (0.68) a	2.04 (0.73)	2.37 (1.00)	5.52 (2176)	0.005	0.059
9	Constant observation	1.98 (0.84)	1.99 (0.89)	2.08 (0.81)	1.87 (0.79)	0.75 (2175)	0.473	
7	PICU	3.58 (1.17)	3.36 (1.23) a	3.62 (1.04) c	3.91 (1.15)	19.79 (2177)	0.000	0.183
11	Open-area seclusion	2.15 (0.81)	1.96 (0.72) a	2.11 (0.67) c	2.50 (0.98)	6.96 (2176)	0.001	0.073
2	Physical restraint	2.59 (1.00)	2.51 (1.02)	2.66 (1.00)	2.65 (0.97)	0.459 (2176)	0.633	
6	IM medication	2.71 (1.04)	2.73 (1.14)	2.77 (0.99)	2.62 (0.92)	0.29 (2177)	0.746	
4	Seclusion	3.02 (1.09)	2.65 (0.98) a	3.00 (1.02) c	3.68 (1.05)	15.45 (2177)	0.000	0.149
8	Mechanical restraint	3.58 (1.17)	2.36 (1.23) a	3.62 (1.04)	3.91 (1.15)	3.37 (2176)	0.037	0.037
10	Net bed	3.91 (1.03)	3.65 (1.08) a	4.04 (0.90) c	4.21 (1.00)	5.29 (2176)	0.006	0.057
HCP								
1	PRN (as-needed) medication	1.30 (0.52)	1.30 (0.48)	1.45 (0.60) c	1.17 (0.44)	8.51 (2361)	0.000	0.045
3	Intermittent observation	1.36 (0.60)	1.34 (0.54)	1.38 (0.66)	1.38 (0.66)	0.246 (2361)	0.782	
5	Time out	1.66 (0.77)	1.41 (0.60) a	1.62 (0.75) c	2.02 (0.86)	21.46 (2361)	0.000	0.106
9	Constant observation	1.72 (0.80)	1.66 (0.82)	1.77 (0.81)	1.73 (0.77)	0.56 (2361)	0.571	
7	PICU	2.29 (1.14)	2.06 (0.95) a,b	1.70 (0.73) c	3.19 (1.17)	72.63 (2360)	0.000	0.287
11	Open-area seclusion	2.48 (1.13)	2.05 (0.94) a,b	2.58 (1.15) c	2.93 (1.14)	21.78 (2359)	0.000	0.108
2	Physical restraint	2.12 (0.90)	2.38 (0.98) a,b	2.06 (0.80)	1.85 (0.82)	11.42 (2360)	0.000	0.060
6	IM medication	2.03 (0.77)	2.12 (0.72) a	2.22 (0.82) c	1.70 (0.68)	15.41 (2359)	0.000	0.079
4	Seclusion	2.47 (1.15)	1.89 (0.73) a	2.00 (0.71) c	3.72 (0.99)	184.99 (2361)	0.000	0.506
8	Mechanical restraint	3.31 (1.18)	2.61 (0.93) a	2.72 (0.98) c	4.24 (0.88)	110.62 (2361)	0.000	0.380
10	Net bed	4.22 (0.95)	3.97 (1.07) a	4.03 (0.86) c	4.76 (0.56)	29.34 (2361)	0.000	0.140

PRN Pro re nata, PICU Psychiatric intensive care unit, IM Intramuscular, NOK Next-of-kin, HCP Healthcare professionals

^a^Muensingen vs. Monthey, *p* < 0.05, Sidac post-hoc

^b^Muensingen vs. Zurich, *p* < 0.05, Sidac post-hoc

^c^Monthey vs. Zurich, *p* < 0.05, Sidac post-hoc

Patients from Monthey appeared to have higher acceptance for measures like PRN and intermittent observation than those from Zurich and Muensingen. In contrast, we found lower acceptance for seclusion in this group (significant difference between Monthey and Zurich). Physical restraint again met significantly higher acceptance from Monthey’s patients compared to the other two study sites. Also, for intramuscular medication (used in all study sites) this trend could be shown. The study site did not account for much of the variance and explained up to 6% (in PRN) of the variance in this group.

In NOK we found a comparable trend for PRN and intermittent observation (higher acceptance in Monthey). The NOK in Monthey agreed significantly lower to PICU, open area seclusion, seclusion, and the net bed compared to those from the other study sites. For mechanical restraint significant differences appeared between Muensingen and Monthey (lower acceptance in Monthey). The study site accounted for a variance of up to 15% in seclusion and 18% in PICU.

The most diverse picture appeared for the HCP’s attitudes. We found comparable ratings only in the attitude on intermittent and constant observation. PRN, and interestingly IM medication and physical restraint were higher accepted in Monthey. In contrast, open area seclusion met significantly higher acceptance in Muensingen compared to the other two study sites. Seclusion, mechanical restraint and the net bed were significantly lower accepted from HCP in Monthey compared to the other two study sites. In HCP we found the highest variances according to the study sites, with up to 50% for seclusion (for details see Table 4).

### Relationship between experience/usage and attitudes towards containment measures

Reported experience or use of containment measures largely differed across the examined measures and also varied among group membership and the study sites (Table 5). However, for all groups, a strong negative relationship between experience or usage and attitudes could be found (rs = −.70, *p* < .001). Higher experience was mostly linked to higher agreement. Correlations were higher for Monthey (r_s patients_ = −.82, *p* < .05; r_s NOK_ = −.74, p < .05; r_s HCP_ = −.84, p < .05) than for Zurich (r_s patients_ = −.76, p < .05; r_s NOK_ = −.74, p < .05; r_s HCP_ = −.77, p < .05) and Muensingen (r_s patients_ = −.73, p < .05; r_s NOK_ = −.55, n.s.; r_s HCP_ = −.41, n.s.)

**Table 5:** Percentage of reported experience or use of containment measures, separated by groups and study sites

		Patients			NOK			HCP		
		Muensingen	Zurich	Monthey	Muensingen	Zurich	Monthey	Muensingen	Zurich	Monthey
1	PRN (as-needed) medication	72%	75%	85%	56%	73%	66%	87%	87%	62%
3	Intermittent observation	41%	42%	49%	25%	61%	51%	72%	84%	82%
5	Time out	30%	32%	43%	13%	35%	36%	78%	79%	61%
9	Constant observation	24%	19%	17%	11%	15%	28%	57%	84%	81%
7	PICU	48%	57%	18%	66%	62%	34%	93%	92%	49%
11	Open-area seclusion	25%	14%	15%	17%	12%	17%	31%	16%	30%
2	Physical restraint	14%	28%	21%	9%	13%	21%	50%	66%	76%
6	IM medication	18%	17%	22%	11%	9%	43%	69%	72%	86%
4	Seclusion	33%	40%	17%	25%	37%	19%	86%	86%	43%
8	Mechanical restraint	28%	16%	11%	17%	14%	20%	83%	76%	45%
10	Net bed	1%	0%	2%	0%	0%	7%	1%	1%	0%
	Mean	30%	31%	27%	23%	30%	31%	64%	68%	56%

*NOK* Next-of-kin, *HCP* Healthcare professionals, *PRN* Pro re nata, *PICU* Psychiatric intensive care unit, *IM* Intramuscular

## Discussion

This study showed that the attitudes towards containment measures significantly varied among the three study sites, located in different states in Switzerland. These differences between the study sites appeared in all three participating groups (patients, NOK and HCP) but were more evident in the HCP, followed by NOK and then patients which appeared to have the smallest study site dependent differences in their attitudes. The study site accounted for a variance from nearly zero in PRN, time out, intermittent and constant observation to 15% in seclusion. In those containment measures which found more agreement, the variance between the study sites was smaller.

Therefore, we can confirm our hypothesis that the study site accounts for some of the variance in the attitudes towards containment measures. We were also able to show that HCP show higher approval for containment measures if they are exerted at their study site. In patients and NOK there was no such, or a smaller effect. Thus, for patients and NOK, we had to reject our hypothesis, that the experience of specific measures is associated with a higher approval. The good internal consistency with a Cronbach alpha =0.83 in our study was in line with other studies [32, 33] and allowed the assessment of a global attitude value which also differed significantly between the study sites.

The traditions and policies of Monthey are different to Muensingen and Zurich according to a 50-year history of an open ward policy and abolition of seclusion and mechanical restraint. The approval of HCP towards these measures was significantly lower in Monthey, compared to Zurich and Muensingen. This indicates an association between the actual exertion or banishment of coercive measures and the attitudes towards those measures. In NOK and patients, we found the same trend but with smaller effect sizes; especially in patients.

These findings go in line with former studies which outlined that besides patients’ characteristics clinic traditions and policies have a relevant impact on the actual usage of coercion [34]. In Monthey, the abolishment of seclusion and restraint and the implementation of an open ward policy are viewed as an achievement. HCP of this clinic agree to this policy and thus, might have a rather negative attitude towards the abolished measures compared to HCP of the other study sites, where seclusion and mechanical restraint are still part of a “clinical routine” if coercion is needed. On one hand, HCP might be influenced by the policy and training of the working place. On the other hand, the attitude of a person might influence the choice of the working place. Nevertheless, compared to the less invasive measures, the approval rate for seclusion and mechanical restraint was low also in HCP from Zurich and Muensingen. This underlines the general disagreement with coercion in all study sites.

Compared to HCP in which the study site explains a relevant part of the variance in the attitudes, the patients’ attitudes were more homogeneous. Patients in Zurich and Muensingen had more negative attitudes compared to HCP or NOK and thus, had more similar attitudes with patients in Monthey. In the least approved measures (net bed, mechanical restraint and IM medication) no significant differences were found between the study sites. For seclusion we found significantly less agreement in Monthey compared to Zurich. This finding goes in line with another study showing that on wards where more seclusion was used, the patients’ agreement with this measure was higher compared to wards where less seclusion was used [22]. Nevertheless, patients who experienced seclusion described feelings of humiliation or of being punished, and a lack of information why they had been secluded [35, 36]. Also one study found that seclusion accounted for 46% of the explained variance of perceived coercion [37].

Compared to the other two study sites, patients (and HCP) in Monthey agreed significantly higher with IM medication. This may be caused by the fact that in Zurich and Muensingen the application of coercive medication is mostly associated with a lower accepted coercive measure (e.g. seclusion or restraint). In contrast, in Monthey only physical restraint (in our study higher agreement compared to seclusion and restraint) can be applied in combination with coercive medication.

Our results reveal the complexity in which the usage of coercive measures is embedded. It is important to gain a more comprehensive understanding on characteristics of coercive situations and their effect on the perception of coercion in patients. In this context the most important finding of this study seems to be that the differences in the attitudes are more pronounced in HCP and contribute for up to 50% of the variance in the HCP’s attitudes. The smaller differences in patients suggest a more homogenous view in this group. Patients might be not that much influenced by clinic traditions and policies.

We found that patients, independent of the study site, showed the least approval for the net bed and mechanical restraint. In Zurich and Muensingen these measures were followed by IM medication and then seclusion whereas in Monthey seclusion was followed by IM medication. Although the patient group is more homogenous it is not possible to define one coercive measure which is “most approved” by patients in general. Personal experience seems to have an impact on the attitudes towards these measures. It should be the aim of further studies to assess to what extend personal experiences but also specific traditions and policies of a clinic influence the patients’ attitudes towards specific measures. These aspects should be taken into account when the decision for or against a specific measure is made. The usage of coercion is dilemmatic in many aspects and there is not one right and especially no generalizable answer to meet this dilemma. We therefore draw differing conclusions compared to a previous study [38] and state that, for the individual patient it is relevant which of the diverse containment and especially of the coercive measures is exerted. In the decision-making process which containment measure to use it is important to bear in mind that as a HCP of a specific clinic one might be influenced by its clinic traditions and policies. Whereas, in patients these aspects have less influence and individual experiences and attitudes seem to be more important. Thus, the patient should be included in the decision which measure is used whenever this is possible. In acute and maybe endangering situations an expedient negotiation with the patient is sometimes unfeasible. Meeting these problems, advance directives were shown to be successful [39]. If a coercive measure has to be used, information about the following steps and the patients ‘rights should be provided by the HCP and the autonomy of the patient should be maintained whenever possible (e.g. allowing visits of NOK, phone calls or mails) [40]. A specific debriefing session with the patient has also proven to be successful to reduce traumatic experiences [1, 41]. This therapeutic strategy may help affected patients to frame the measure in the context of a treatment which aims to encourage their personal recovery. Coercion was shown to reduce the treatment satisfaction. Therefore, efforts should be made to strengthen the therapeutic relationship if it comes to its usage [42].

### Limitations

The participation in this study was voluntary and only a small part of those patients who were treated during the study period participated in the study. Unfortunately, it is not clear how many patients refused to participate. The study design did not allow for a structured comparison of those patients who were invited and those who participated/refused. Furthermore, the assessment of the NOK was anonymized, and our data do not allow for a comparison of those NOK who participated/refused. Thus, the results might be biased in patients and their NOK. The response rate of HCP differed between the study sites with a high response rate in Monthey, followed by Muensingen and the lowest response rate in Zurich. Participants were sufficiently motivated to complete the voluntary survey, and as a result, individuals with strong views regarding containment measures were probably over-sampled. A refusal to participate in the study could have been caused by the patients’ or their NOK’s disapproval with the treatment. We did not assess factors like symptom severity and thus were not able to correct for these possible influencing factors. Of all participating patients, 21% were involuntarily hospitalized which is comparable to the total rates of involuntary hospitalization at the three study sites in general. Also, the patients did not differ in the distribution of their diagnoses from the whole sample treated at the study sites. The distribution of the different professions (mental health nurses, psychiatrists and psychologists) who completed the questionnaire is comparable to the distribution on the different wards included in the study. As a consequence, we can assume that, despite of the limitations, the participants might be representative for the whole sample at the three study sites.

The ACMQ does only ask for the attitude and thus, we were not able to assess why participants agreed with some and disagreed with other measures. We also do not know when those participants with experiences were exposed to a specific measure. This might also have led to a bias, as former studies have shown that the time between experience of a coercive measure and the assessment influences the approval of the measure [43].

Due to the different languages at the study sites (German in Zurich and Muensingen, French in Monthey) the German version was translated into a French version by members of the study team (T.R. and G.K.). They speak German and French fluently and therefore the chance of imprecisions and biased results due to language differences is minimal.

We were able to show that there are differences in the attitudes towards containment measures and in clinic traditions and policies between the three study sites. Nevertheless, we did not use a structured instrument to assess the clinic traditions and policies and its influence on participants and we cannot clearly deduce the factors which contribute to this cultural difference. Besides the traditions in psychiatric care, sociocultural aspects like cultural and political views, but also organizational factors like different training programs in the prevention of aggression and coercion, the patient/HCP ratio and the turn-over rate of patients at the three study sites may have influenced the attitudes of the participants.

Analyses were confined to responses from Swiss participants and their beliefs and attitudes may differ to other countries. Nevertheless, the study population was big, and the statements of the participants were congruent with data on the usage of coercion which we extracted from the clinical routine documentation.

## Conclusions

The study site accounts for a substantial proportion of the variance between the attitudes towards containment measures - especially in HCP. The differences in the attitudes may be partly explained by the differing clinic traditions and policies at the three study sites (e.g. different training-approaches of health care professionals in de-escalation techniques, values and the lack of standard operating procedures). The different attitudes were especially expressed in HCP, whereas in patients the attitudes vary less within the different study sites. This finding emphasizes the importance to adjust the usage of containment measures and especially of coercion on the patients’ attitudes and, if necessary, apply those measures which they agree more with. Advanced directives and treatment planning might be helpful tools to capture the individual attitudes of a patient. Future studies should emphasize to gain more knowledge about the factors which shape the attitude towards specific measures.

## Abbreviations

ACMQ: Attitude to Containment Measures Questionnaire
HCP: Healthcare professionals
IM: Intramuscular
NOK: Next of kin
PICU: Psychiatric intensive care unit
PRN: Pro re nata (as needed)
